# And the Dog Was Barking: Transforming Quality of Life in Diabetes Through Innovative Hypoglycemia Detection

**DOI:** 10.1111/1753-0407.70143

**Published:** 2025-08-12

**Authors:** Theocharis Koufakis, Djordje S. Popovic, Nikolaos Papanas

**Affiliations:** ^1^ Second Propaedeutic Department of Internal Medicine Hippokration General Hospital, Aristotle University of Thessaloniki Thessaloniki Greece; ^2^ Clinic for Endocrinology Diabetes and Metabolic Disorders, Clinical Centre of Vojvodina Novi Sad Serbia; ^3^ Medical Faculty University of Novi sad Novi Sad Serbia; ^4^ Diabetes Centre, Second Department of Internal Medicine Democritus University of Thrace, University Hospital of Alexandroupolis Alexandroupolis Greece

**Keywords:** continuous glucose monitoring, hypoglycemia, quality of life, sensor‐augmented insulin pumps

## 1 Hypoglycemia: A Limiting Factor in Diabetes Management

For people living with diabetes, hypoglycemia has long been a silent threat—its approach often as unnoticed as a dog barking in the distance, unheard by those most at risk. Since the earliest clinical descriptions of diabetes, hypoglycemia has been recognized as a critical and sometimes life‐threatening complication of glucose‐lowering therapy [1]. In the early 20th century, with the advent of insulin therapy, hypoglycemia emerged as a new and immediate concern, often identified only after the onset of severe neuroglycopenic symptoms. Early detection relied primarily on clinical observation and patient self‐reporting of warning signs, with little objective measurement available. The development of capillary blood glucose testing in the 1970s and 1980s marked a major advance, enabling both patients and clinicians to more accurately identify and document hypoglycemic episodes. Over the past two decades, the evaluation of hypoglycemia has been revolutionized by continuous glucose monitoring (CGM) technologies and the integration of predictive algorithms, which now allow for real‐time detection, detailed glycemic profiling, and proactive management [2]. This historical progression from symptom‐based recognition to advanced digital monitoring reflects the ongoing commitment to improving safety and outcomes in diabetes care.

However, hypoglycemia remains a principal barrier to optimal glycemic control in diabetes, particularly for individuals with type 1 diabetes mellitus (T1DM), who require intensive insulin regimens to prevent microvascular and macrovascular complications [3]. While intensified therapy reduces long‐term risks, it simultaneously increases the incidence of hypoglycemia, necessitating careful therapeutic balancing. A significant subset of individuals with T1DM—estimated at up to 25%—develop hypoglycemia unawareness, where autonomic warning symptoms such as sweating and tremor become attenuated or absent [4]. This phenomenon greatly elevates the risk for severe events requiring external assistance, often forcing patients and clinicians to accept higher glucose targets than recommended [5]. Thus, the fear and reality of hypoglycemia continue to restrict the full benefits of modern diabetes therapies and remain a central concern in clinical care.

The implications of hypoglycemia extend well beyond transient physical symptoms. Acute episodes have been associated with a heightened risk of cardiac arrhythmias and myocardial ischemia, linked to autonomic surges and altered cardiac repolarization [6]. Hypoglycemia may also induce a prothrombotic state, increasing platelet activation and coagulation, thereby further raising cardiovascular risk—an especially pertinent issue in people already predisposed to vascular disease [7]. At a neurological level, repeated or severe events can result in cognitive dysfunction, seizures, and, rarely, irreversible brain injury [8]. Importantly, the psychological toll is substantial: persistent fear of hypoglycemia can drive suboptimal self‐management, such as intentional hyperglycemia, and contribute to reduced quality of life [9]. Fear of hypoglycemia is a multidimensional phenomenon, involving not only anxiety about experiencing the physical symptoms of hypoglycemia but also apprehension regarding the unpredictability of such episodes, potential social embarrassment, and the risk of harm to oneself or others. As highlighted by Bloomgarden [10], this fear can create a significant emotional burden, leading patients to consciously avoid optimal glycemic targets in order to minimize perceived risk, which paradoxically increases the likelihood of long‐term diabetes complications. The need for improved detection and prevention strategies is therefore both medical and psychosocial in nature.

## 2 Preventing Hypoglycemia: Traditional and Novel Tools in Our Arsenal

An intriguing example of non‐technological innovation in this field is the use of diabetes alert dogs (DADs), often Labradors, trained to detect specific scent markers—volatile organic compounds—in the breath or sweat of individuals during hypoglycemic episodes [11]. Controlled studies and real‐world experience suggest that DADs can provide early warnings before symptomatic awareness; facilitating more rapid intervention [12]. Furthermore, beyond physiological safety, the presence of a trained service dog offers meaningful psychosocial benefits, including reduced anxiety and increased independence, especially for children and those experiencing nocturnal hypoglycemia [13]. While challenges remain in standardization and evidence quality, DADs exemplify the value of integrating biological detection with patient‐centered care.

Technological advances in CGM have transformed the landscape of hypoglycemia detection and prevention. CGM systems provide near‐continuous, subcutaneous glucose data, delivering real‐time alerts for actual or impending hypoglycemia. Numerous randomized controlled trials have shown that CGM significantly reduces the frequency and severity of hypoglycemia, especially in those with impaired awareness, while also supporting improved glycemic control [14]. CGM also enables trend analysis and more precise therapy adjustments, thus empowering patients and clinicians to optimize diabetes management and mitigate risk [15].

Sensor‐augmented insulin pumps (SAPs) with low glucose suspend (LGS) and predictive low glucose suspend (PLGS) functions represent another major step forward. By integrating CGM data, these devices can automatically halt basal insulin infusion when low or dropping glucose is detected or predicted, thereby reducing the risk of severe hypoglycemia, particularly overnight. Large trials, including the ASPIRE and SMILE studies, have confirmed that SAPs with LGS/PLGS significantly decrease hypoglycemia burden without compromising overall glycemic targets, benefiting high‐risk groups such as those with hypoglycemia unawareness [16, 17].

The latest generation of CGM and SAP devices employ artificial intelligence (AI) and machine learning to predict hypoglycemic events before they occur. AI algorithms continuously analyze large streams of glucose data, insulin dosing, and contextual factors in real time, identifying patterns and predicting when blood glucose is likely to fall below a safe threshold. In CGM systems, this can trigger timely alerts to the patient or caregiver before hypoglycemia actually develops, allowing for preventive action. In SAPs and closed‐loop systems, AI not only predicts impending hypoglycemia but can automatically adjust or suspend insulin delivery to avert the event. These predictive and automated features are especially valuable at night when patients are most vulnerable and may not be aware of symptoms. Preliminary clinical studies indicate that such predictive technologies can substantially reduce nocturnal hypoglycemia and improve user confidence while the accuracy and personalization of these algorithms continue to advance [18, 19]. As AI‐driven systems are integrated into closed‐loop insulin delivery, their potential for individualized diabetes management is expected to expand further.

## 3 What Does the Next Chapter in Diabetes Care Hold?

Non‐invasive glucose monitoring using sweat, saliva, or transdermal technologies holds significant promise for the future, aiming to reduce the burden of needle‐based monitoring and increase adherence. In parallel, wearable health devices—such as smartwatches—are being developed to provide multi‐parameter physiologic data, enabling more robust and context‐aware hypoglycemia prediction [20]. The evolution of closed‐loop “artificial pancreas” systems, which automatically adjust insulin delivery based on real‐time sensor data, is moving diabetes management closer to seamless automation, reducing both hypo‐ and hyperglycemic excursions [21]. The integration of biosensors, digital health solutions, and AI‐driven analytics is expected to herald a new era of proactive, individualized diabetes management.

In conclusion, recent innovations in hypoglycemia detection—spanning from the keen instincts of trained dogs to advanced CGMs, SAPs, and AI—have redefined diabetes management. These technologies have not only reduced the risk and burden of hypoglycemia, but also alleviated its psychological consequences, enhancing quality of life and patient autonomy. Where once individuals relied on a dog's bark for protection, today's solutions ensure that warnings are timely, precise, and reliable. For many years, the dog was barking in the middle of the night; at last, now we can enjoy better sleep.

## Author Contributions

T.K. reviewed the literature and wrote the first version of the manuscript. D.S.P. and N.P. reviewed the literature and edited the manuscript. All authors have read and approved the final version of the manuscript.

## Conflicts of Interest

T.K. is an Editorial Board member of *Journal of Diabetes* and a co‐author of this article. To minimize bias, they were excluded from all editorial decision‐making related to the acceptance of this article for publication. T.K. has received honoraria for lectures from AstraZeneca, Sanofi, Boehringer Ingelheim, Pharmaserve Lilly, Menarini, and Novo Nordisk, for advisory boards from Novo Nordisk, Roche, Sanofi, and Boehringer Ingelheim, and has participated in sponsored studies by Eli‐Lilly, AstraZeneca, and Novo Nordisk. D.S.P. declares associations to Abbott, Alkaloid, AstraZeneca, Boehringer‐Ingelheim, Berlin‐Chemie, Eli Lilly, Galenika, Krka, Merck, Novo Nordisk, PharmaSwiss, Sanofi‐Aventis, Servier, Viatris, ADOC Pharma, and Worwag Pharma. N.P. has been an advisory board member of TrigoCare International, Abbott, AstraZeneca, Elpen, MSD, Novartis, Novo Nordisk, Sanofi Aventis, and Takeda; has participated in sponsored studies by Eli Lilly, MSD, Novo Nordisk, Novartis, and Sanofi Aventis; received honoraria as a speaker for AstraZeneca, Boehringer Ingelheim, Eli Lilly, Elpen, Galenica, GSK, MSD, Mylan, Novartis, Novo Nordisk, Pfizer, Sanofi Aventis, Takeda, and Vianex; and attended conferences sponsored by TrigoCare International, AstraZeneca, Boehringer Ingelheim, Eli Lilly, GSK, Novartis, Novo Nordisk, Pfizer, and Sanofi Aventis.
